# Myeloid Cells in Circulation and Tumor Microenvironment of Colorectal Cancer Patients with Early and Advanced Disease Stages

**DOI:** 10.1155/2020/9678168

**Published:** 2020-06-12

**Authors:** Salman M. Toor, Sarah Khalaf, Khaled Murshed, Mohamed Abu Nada, Eyad Elkord

**Affiliations:** ^1^Cancer Research Center, Qatar Biomedical Research Institute (QBRI), Hamad Bin Khalifa University (HBKU), Qatar Foundation (QF), Doha, Qatar; ^2^College of Health & Life Sciences (CHLS), Hamad Bin Khalifa University (HBKU), Qatar Foundation (QF), Doha, Qatar; ^3^Department of Pathology, Hamad Medical Corporation, Doha, Qatar; ^4^Department of Surgery, Hamad Medical Corporation, Doha, Qatar

## Abstract

Myeloid-derived suppressor cells (MDSCs) are a heterogenous population of cells that have been implicated in the development of an immunosuppressive environment, which promotes tumorigenesis and tumor progression. Numerous studies have reported expansion of MDSCs in circulation and the tumor microenvironment (TME) of cancer patients. However, due to the heterogenic nature of MDSCs and the different approaches for their identification, their detailed characterization and impact on disease progression in cancer patients are warranted. In this study, we investigated the levels of different myeloid cell subsets and antigen-presenting cells (APCs) using flow cytometry in unfractionated whole blood (WB), peripheral blood mononuclear cells (PBMCs), tumor tissue (TT), and adjacent normal tissue (NT) of colorectal cancer (CRC) patients. We found high levels of granulocytic myeloid cells (GMCs) in whole blood, but their levels were significantly lower in PBMCs. Importantly, we found significantly higher levels of GMCs in the TME compared to NT. In addition, monocytic myeloid cells (MMCs) showed significantly higher levels in PBMCs of CRC patients, compared to healthy donors (HDs). Notably, patients with advanced disease stages showed significantly higher levels of GMCs compared to early stages in whole blood, but PBMCs and tumor-infiltrating myeloid cells did not show any significant differences. Lastly, we found that levels of GMCs decreased, while IMCs increased in the TME with tumor budding. Our results highlight the importance of investigating the levels of different myeloid cell subsets in PBMCs versus whole blood of cancer patients and improve current knowledge on the potential prognostic significance of myeloid cells in CRC patients.

## 1. Introduction

Immunosuppression is recognized as a key factor in driving tumorigenesis [1]. Cancer cells constantly evolve to evade immune destruction and promote tumor growth and progression by exploiting several immune evasive mechanisms. These mechanisms include induction of immunosuppressive cells such as regulatory T cells (Tregs), myeloid-derived suppressor cells (MDSCs), M2 macrophages and upregulation of coinhibitory immune checkpoint molecules for attenuation of tumor-reactive T cells in the tumor microenvironment (TME) [2, 3].

Expansion of MDSCs in circulation and TME of cancer patients has been widely reported. Their accumulation and activation have been shown to correlate with tumor progression, metastasis, and relapse of several human cancers [4], and negatively correlate with efficacy of immunotherapy [5]. MDSCs consist of a heterogeneous population of myeloid cells at various stages of maturation, which originated from hematopoietic progenitor cells and possess potent immunosuppressive activity [6]. Accumulating evidences have led to basic phenotypic classification of MDSCs as cells that express CD33 and CD11b myeloid markers but lack HLA-DR (MHC-class-II) expression [7]. MDSCs can be further categorized into two main subsets termed polymorphonuclear (PMN-MDSC) or granulocytic (G-MDSCs) and monocytic (M-MDSCs). These cell subsets are phenotypically and morphologically similar to mature neutrophils and monocytes, respectively. G-MDSCs can be defined as CD33^+^CD11b^+^HLA-DR^−/low^CD14^−^CD15^+^, and M-MDSCs as CD33^+^CD11b^+^HLA-DR^−/low^CD14^+^CD15^−^ [8]. M-MDSCs can be phenotypically distinguished from monocytes by lack of HLA-DR expression, while identification of other MDSC subsets from different myeloid populations may require investigating additional markers or confirmation on their suppressive abilities [9]. A more recent, additional subset has been identified in a number of studies, termed immature or early-stage MDSCs (e-MDSCs), identified as CD33^+^CD11b^+^HLA-DR^−/low^CD14^−^CD15^−^ [8, 10]. MDSCs exert their immunosuppressive influence through various immunosuppressive factors, which include release of arginase-1, nitric oxide (NO), inducible nitric oxide synthase (iNOS), reactive oxygen species (ROS), and reactive nitrogen species (RNS) [6].

Colorectal cancer (CRC) is the third most common cancer and the fourth main cause of all cancer-related deaths globally [11]. Extensive ongoing research is aimed at improving survival rates of CRC patients. Recent developments in cancer immunotherapy have focused on modulating the activity of tumor-infiltrating cytotoxic T-lymphocytes (CTLs) via blocking coinhibitory immune checkpoint molecules in the TME. Importantly, studies have shown that in CRC patients, the tumor mutational landscape directly influences effective antitumoral immune responses, as microsatellite instable and mismatch repair(MMR) deficient tumors respond better to immunotherapy [12]. However, the presence of an immunosuppressive network within the TME greatly limits the efficacy of checkpoint blockade and also contributes to acquired resistance to therapy [13]. Therefore, investigations on immunosuppressive cells in CRC patients are warranted to identify potential contributors of resistance and targets for effective therapies.

In this study, we compared the levels of different myeloid cell subsets in periphery and the TME of CRC patients. We investigated differences between levels of different myeloid cell subsets in whole blood and PBMCs to highlight the significance of the peripheral source (e.g. whole blood versus PBMC). Importantly, we investigated the differences between levels of myeloid cells in CRC patients with their clinicopathologic features, tumor node metastasis (TNM) disease staging, and degrees of tumor budding, to indicate their potential roles in disease progression.

## 2. Materials and Methods

### 2.1. Sample Collection and Study Populations

Fresh whole blood (WB) samples were collected from 88 treatment-naïve CRC patients, and tumor tissues (TT) and paired, adjacent non-cancerous normal tissues (NT) were collected from 31 out of the 88 CRC patients, who undertook surgery at Hamad Medical Corporation, Doha, Qatar. Buffy coats were collected from healthy individuals/controls (*n* = 25), obtained from the Blood Donor Center at Hamad Medical Corporation Doha, Qatar. Characteristic features of the study populations are shown in Table 1. All participants provided informed written consent prior to sample collection. This study was approved by the Qatar Biomedical Research Institute, Doha, Qatar (Approval no. 2018-018), and Hamad Medical Corporation, Doha, Qatar (Approval no. MRC-02-18-012), institutional review boards and was performed in accordance with applicable guidelines and regulations.

Peripheral blood mononuclear cells (PBMCs) were isolated from fresh whole blood and buffy coats by density-gradient centrifugation using Histopaque-1077 (Sigma-Aldrich, St. Louis, MO, USA). PBMCs and tissue specimens were frozen in freezing media (10% dimethyl sulfoxide, 50% fetal calf serum, and 40% RPMI-1640 medium) and stored in liquid nitrogen to be used in batches in subsequent analyses.

### 2.2. Preparation of Single-Cell Suspensions from Tissue Samples

Cell suspensions were prepared from NT and TT by mechanical dissociation using the gentleMACS Dissociator (Miltenyi Biotec, Bergisch Gladbach, Germany), without using enzymes as previously described [14]. Briefly, tissues stored in freezing media were thawed and washed with phosphate-buffered saline (PBS) and added to serum-free RPMI-1640 media. A surgical scalpel was used to mechanically cut tissue into small pieces (~2-4 mm). Tissue pieces were added to gentleMACS Dissociator C tubes for dissociation into single-cell suspensions. A 100 *μ*M cell strainer was used to remove aggregates and debris from the resulting cell suspension. The cell suspension was washed with PBS and stained for flow cytometric analyses.

### 2.3. Immunophenotyping Using Multiparametric Flow Cytometry

Fresh whole blood staining was performed using 100 *μ*l of blood for each sample, as previouly described [15]. Briefly, FcR Blocking Reagent (Miltenyi Biotec) was added first to block Fc receptors. Antibodies for myeloid markers (anti-human CD33-Fluorescein Isothiocyanate (FITC) (clone HIM3-4), CD11b-Allophycocyanin/Cyanine7 (APC-Cy7) (clone ICRF44), HLA-DR-phycoerythrin (PE) (clone G46-6), CD14-PE-Cy7 (clone M5E2), and CD15-APC (clone HI98)) (all from BD Biosciences, Oxford, UK) were added, and tubes were incubated at 4°C for 30 min. RBC lysis was performed using BD FACS lysing solution (BD Biosciences), as per the manufacturer's protocol. Cells were washed twice with PBS and resuspended in flow cytometry staining buffer (PBS + 1%FCS + 0.1%sodium azide).

Single-cell suspensions from tissues were washed with PBS, and the FcR Blocking Reagent (Miltenyi Biotec) was added, followed by staining with the aforementioned antibodies for myeloid markers. 7-AAD Viability Staining Solution (BioLegend, San Diego, USA) was used to gate live cells. Cells were incubated at 4°C for 30 min and then washed twice with PBS, followed by resuspension in flow cytometry staining buffer.

Data were acquired on a BD LSRFortessa X-20 SORP flow cytometer using BD FACSDiva software (BD Biosciences), and analyses were performed on FlowJo V10 software (FlowJo, Ashland, USA).

### 2.4. Statistical Analyses

Statistical analyses and representations were performed using GraphPad Prism 8 software (GraphPad Software, California, USA). The Shapiro-Wilk normality test was used to test for normality of datasets. Various tests for statistical significance were then applied; one-way ANOVA for multiple comparisons, paired *t*-test for paired samples with a normal distribution, and Wilcoxon matched-pairs signed rank for paired samples that did not follow normal distribution. For unpaired samples, an unpaired *t*-test was applied for normally distributed datasets and Mann-Whitney for samples that did not follow a normal distribution. A *P* value of >0.05 was considered statistically nonsignificant. The *P* values are represented as follows: ^∗∗∗^*P* < 0.001, ^∗∗^*P* < 0.01, and ^∗^*P* < 0.05. Levels of cells were analyzed and compared as relative percentages from the parent population(s). All values are presented as the mean ± standard error of the mean (SEM).

Whole blood analyses were performed on 88 patients. PBMC analyses were performed on 55 patients, and analyses on tissues were performed on 31 out of the total 88 patients. Sample size for each dataset is specified in the figure legends.

## 3. Results

### 3.1. Levels of Circulating Myeloid Cells in CRC Patients

**Figure 1:**
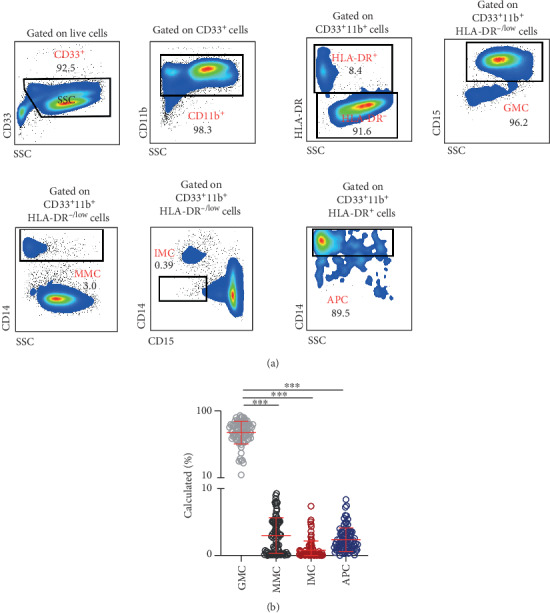
Levels of myeloid cells in whole blood of CRC patients. Peripheral whole blood samples obtained from patients with colorectal cancer (CRC; *n* = 88) were stained for different myeloid cell markers. Representative flow cytometric plots show the gating strategy identifying granulocytic myeloid cells (GMCs; CD33^+^HLA-DR^−^CD15^+^), monocytic myeloid cells (MMCs; CD33^+^HLA-DR^−^CD14^+^), immature myeloid cells (IMCs; CD33^+^HLA-DR^−^CD15^−^CD14^−^), and antigen-presenting cells (APCs; CD33^+^HLA-DR^+^CD14^+^) (a). Cumulative scatter plot shows the calculated percentages of the different myeloid cell subsets in whole blood of CRC patients (b). The *P* values are represented as follows: ^∗∗∗^*P* < 0.001. Data depict the mean ± standard error of the mean (SEM).

We investigated the levels of different myeloid cell subsets in circulation of CRC patients (Figure 1). We referred to them as granulocytic myeloid cells (GMCs; CD33^+^HLA-DR^−^CD15^+^) and immature myeloid cells (IMCs; CD33^+^HLA-DR^−^CD15^−^CD14^−^) due to lack of evidence on their suppressive capabilities to identify them as MDSCs. In addition, we referred to CD33^+^HLA-DR^−^CD14^+^ population as monocytic myeloid cells (MMCs), even though it is primarily comprised of M-MDSCs, for consistency in nomenclature across the various comparisons made in this study. We also investigated the levels of CD33^+^HLA-DR^+^CD14^+^ APCs.

Flow cytometric analyses of fresh whole blood showed GMCs to be the major myeloid subset, compared to the other myeloid cells (GMCs: 70.0 ± 1.6%, MMCs: 3.0 ± 0.3%, IMCs: 0.8 ± 0.1%, and APCs: 2.4 ± 0.2%; Figure 1(b)). However, the majority of these cells include mature neutrophils [16], and the levels of PMN-MDSCs in unfractionated blood are considered to be inaccurate due to the presence of steady-state granulocytes [17].

**Figure 2:**
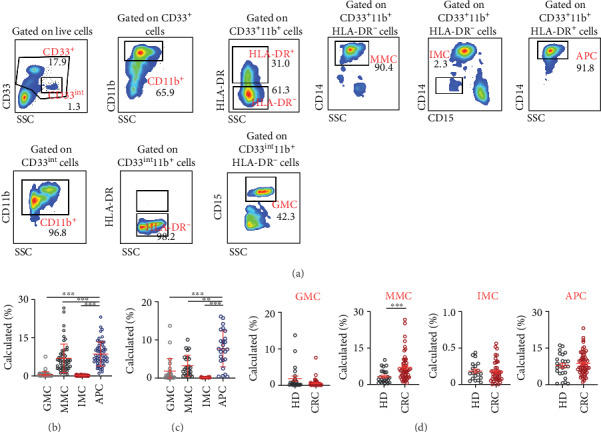
Comparative analyses of levels of myeloid cells in PBMCs of CRC patients versus HDs. PBMCs isolated from peripheral blood of colorectal cancer patients (CRC; *n* = 55) and healthy donors (HDs; *n* = 25) were stained for different myeloid cell markers. Representative flow cytometric plots show the different myeloid cell subsets in PBMC of CRC patients (a). Cumulative scatter plots show the calculated percentages of GMCs, MMCs, IMCs, and APCs in PBMC of CRC patients (b) and HDs (c) and comparisons between CRC patients and HDs (d). The *P* values are represented as follows: ^∗∗∗^*P* < 0.001, ^∗∗^*P* < 0.01. Data depict the mean ± standard error of the mean (SEM).

Therefore, in order to obtain a more robust phenotypical characterization of circulating GMCs, devoid of circulating neutrophils, we isolated PBMCs from whole blood of CRC patients and investigated the levels of the different myeloid cell subsets (Figure 2). There were significantly lower levels of GMCs in PBMCs, compared to their levels in unfractionated whole blood (WB: 70.0 ± 1.6%, PBMCs: 0.6 ± 0.1%; Figure 2(b)). Moreover, contrary to whole blood, MMCs and APCs were highest in PBMCs compared to the other myeloid cell subsets (MMCs: 6.9 ± 0.7%, IMCs: 0.2 ± 0.0%, and APCs: 8.6 ± 0.6%; Figure 2(b)).

### 3.2. CRC Patients Have Higher Levels of Circulating Monocytic Myeloid Cells in PBMCs than Healthy Controls

To determine if the levels of myeloid cells observed in PBMCs from CRC patients were different compared to normal controls, we investigated their levels in healthy individuals (HDs). In healthy controls, APCs were the highest followed by MMCs and GMCs (GMCs: 1.9 ± 0.7%, MMCs: 3.3 ± 0.5%, IMCs: 0.2 ± 0.0%, and APCs: 7.7 ± 0.9%; Figure 2(c)). Comparing CRC patients with healthy controls, CRC patients showed lower levels of GMCs, although not statistically significant (HDs: 1.9 ± 0.7%, CRC: 0.6 ± 0.1%; Figure 2(d)). Importantly, we found that MMCs were significantly higher in CRC patients compared to HDs (HDs: 3.3 ± 0.5%, CRC: 7.4 ± 1.0%; Figure 2(d)). However, IMCs (HDs: 0.2 ± 0.0%, CRC: 0.2 ± 0.0%; Figure 2(d)) and APCs (HDs: 7.7 ± 0.9%, CRC: 8.4 ± 0.6%; Figure 2(d)) showed similar levels in HDs and CRC patients.

### 3.3. Granulocytic Myeloid Cells Accumulate in Tumor Tissues Compared to Normal Tissues of CRC Patients

**Figure 3:**
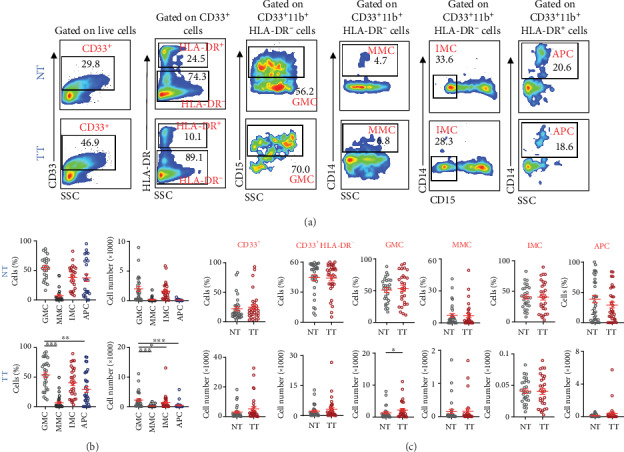
Levels of myeloid cells in normal and tumor tissues of CRC patients. Cell suspensions prepared from normal tissues (NT) and tumor tissues (TT) of CRC patients (*n* = 31) were stained for different myeloid markers. Representative flow cytometric plots show levels of different myeloid cell subsets in NT and TT of CRC patients (a). Cumulative scatter plots show the relative percentages and absolute cell numbers (×1000) of GMCs, MMCs, IMCs, and APCs in NT and TT (b) and comparisons between NT and TT (c). The *P* values are represented as follows: ^∗^*P* < 0.05. Data depict the mean ± standard error of the mean (SEM).

Next, we investigated the levels of different myeloid cell subsets in TT and compared their levels in NT (Figure 3). Our results showed that levels of CD33^+^ and CD33^+^HLA-DR^−^ cell subsets, as relative percentage (cells (%)), and absolute count (cell number), were similar between NT and TT of CRC patients. However, GMCs were significantly higher in TT compared with NT as absolute counts (NT: 1336 ± 372.8, TT: 2202 ± 511.7; Figure 3(c)). MMCs and IMCs showed similar levels in both NT and TT. In addition, absolute counts of APCs showed a higher level in NT compared to TT, although not statistically significant (NT: 113.5 ± 27.0, TT: 382.6 ± 191.4; Figure 3(c)). Moreover, contrary to periphery, the levels of GMCs and IMCs were higher than MMCs in TME.

### 3.4. Granulocytic Myeloid Cells in Whole Blood Were Higher in CRC Patients with Advanced Disease Stages

**Figure 4:**
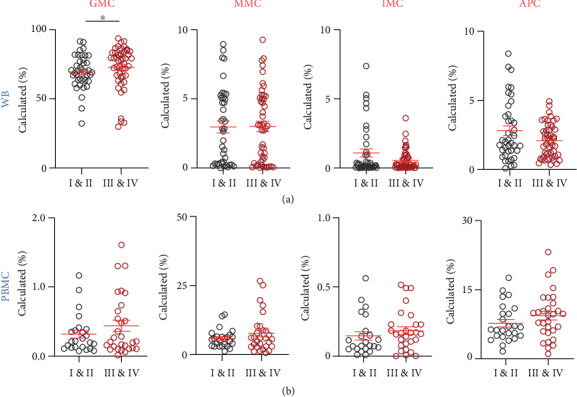
Comparison of levels of circulating myeloid cells in whole blood and PBMCs with TNM disease stages of CRC patients. CRC patient samples were divided into two groups based on the TNM stage, localized disease (stages I and II; WB, *n* = 40 and PBMCs, *n* = 25) and regional lymph node or distant metastases (stages III and IV; WB, *n* = 48 and PBMCs, *n* = 30). Cumulative scatter plots show the differences in calculated percentages of GMCs, MMCs, IMCs, and APCs in WB (a) and PBMCs (b) of CRC patients with different TNM disease stages. The *P* values are represented as follows: ^∗^*P* < 0.05. Data depict the mean ± standard error of the mean (SEM).

Next, we investigated differences between the levels of the different myeloid cell subsets in whole blood and PBMCs with different TNM disease stages. Patients were divided into two groups; stages I and II (localized disease) and stages III and IV (regional lymph node or distant metastases) (Figure 4).

In whole blood, GMCs showed a significant increase in patients with advanced stages (early stages: 69.5 ± 1.9% and advanced stages: 72.9 ± 2.2%; Figure 4(a)). MMCs showed similar levels in both staging groups (early stages: 2.9 ± 0.4% and advanced stages: 3.0 ± 0.4%; Figure 4(a)). However, IMCs showed higher levels in early stages (early stages: 1.0 ± 0.3% and advanced stages: 0.5 ± 0.1%; Figure 4(a)), while APCs showed a higher level in advanced stages (early stages: 2.8 ± 0.3% and advanced stages: 2.1 ± 0.2%; Figure 4(a)), but the data did not show statistical significance.

Interestingly, GMCs remained higher in PBMCs with advanced stages, however, not statistically significant (early stages: 0.3 ± 0.05% and advanced stages: 0.4 ± 0.1%; Figure 4(b)). MMCs also showed a trend of increase in advanced stages (early stages: 5.8 ± 0.6% and advanced stages: 7.7 ± 1.3%; Figure 4(b)), while IMCs (early stages: 0.1 ± 0.03% and advanced stages: 0.2 ± 0.02%; Figure 4(b)) and APCs (early stages: 7.7 ± 0.8% and advanced stages: 9.4 ± 1.0%; Figure 4(b)) showed similar levels in both staging groups.

### 3.5. Differences in the Levels of Tumor-Infiltrating Myeloid Cell Subsets between CRC Patients with Different Disease Stages and Tumor Budding

**Figure 5:**
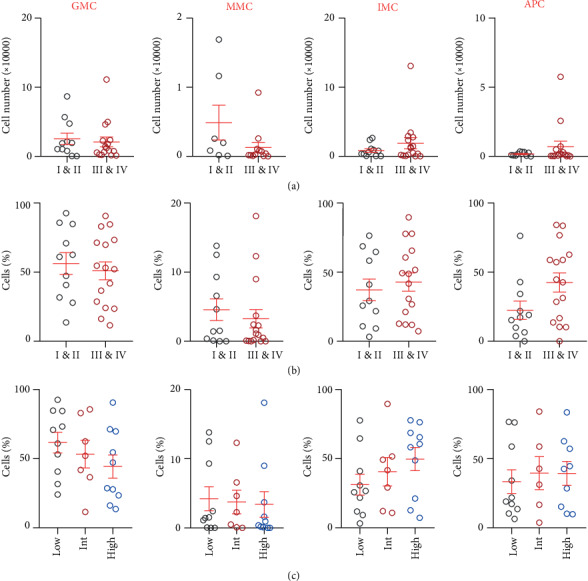
Comparison of levels of tumor-infiltrating myeloid cells with TNM disease stages and tumor budding grades in TT of CRC patients. CRC patient samples were divided into two groups based on TNM stage: localized disease (stages I and II; TT, *n* = 14) and regional lymph node or distant metastases (stages III and IV; TT, *n* = 17) and into three groups based on tumor budding classification (low, *n* = 13; intermediate, *n* = 8; and high, *n* = 10). Cumulative scatter plots show differences in absolute count (a) and relative percentage (b) of GMCs, MMCs, IMCs, and APCs in TT of CRC patients with different TNM stages. Cumulative scatter plots show differences in relative percentage of GMCs, MMCs, IMCs, and APCs in TT of CRC patients with different tumor budding grades (c). Data depict the mean ± standard error of the mean (SEM).

We also investigated differences between the levels of different myeloid cell subsets in the TME with varying TNM disease stages and tumor budding (Figure 5). Interestingly, IMCs showed an opposite trend to that of circulation, with higher levels in advanced stages (early stages: 37.20 ± 7.9% and advanced stages: 42.8 ± 6.6%; Figure 5(b)). GMCs showed a different trend compared with WB as well; early stages showed a higher level (early stages: 56.3 ± 7.9% and advanced stages: 50.9 ± 6.5%; Figure 5(b)). MMCs showed similar levels between the two staging groups (early stages: 4.6 ± 1.6% and advanced stages: 3.3 ± 1.3%; Figure 5(b)). APCs were approximately two times the level observed in early stages compared to advanced stages (early stages: 22.4 ± 6.6% and advanced stages: 42.5 ± 6.9%; Figure 5(b)).

Tumor budding has been proposed as an independent prognostic factor for adverse clinical outcomes in CRC [18]. We divided the patients into three groups based on their tumor budding grades: low, intermediate, and high. Our results showed interesting trends, although not statistically significant (Figure 5(c)). GMCs were lower with higher levels of tumor budding (low: 61.7 ± 7.5%, intermediate: 53.3 ± 9.9%, and high: 44.4 ± 8.4%), while IMCs showed an increasing trend (low: 31.3 ± 7.6%, intermediate: 40.6 ± 10.3%, and high: 49.7 ± 8.4%). MMCs and APCs showed similar levels in all three categories (Figure 5(c)). In addition, 16 patients out of the 31 patients presented with lymphovascular invasion (LVI), which is considered as a vital step in tumor progression towards metastasis [19]. We investigated the levels of different tumor-infiltrating myeloid cells between patients with and without LVI but did not find any significant differences between these two groups (data not shown).

## 4. Discussion

MDSCs have been indicated to promote tumor progression, inhibit antitumor immunity, and hinder various cancer immunotherapies [20, 21]. Elevated MDSC levels have been reported in diverse tumor types [22], which led to the identification of potential clinical targets to block MDSCs in cancer [23]. However, while MDSCs encompass a heterogenous population of suppressive myeloid cells, there is a phenotypical overlap with other cellular populations of myeloid origin. Therefore, it is critical to investigate aberrations in the levels of overall myeloid cells in periphery and TME of cancer patients.

MDSC levels in CRC patients have been reported previously [24, 25]. In the present study, we investigated the levels of different subsets of myeloid cells in whole blood and PBMCs isolated from CRC patients. The difference in densities of PMN-MDSCs and other granulocytes as reported previously provided a rationale for investigating differences in levels of different myeloid cell subsets between whole blood and PBMCs; the variances in myeloid cell subsets between whole blood and PBMCs can potentially differentiate between PMN-MDSCs and GMCs [26]. Neutrophils have high densities, while PMN-MDSCs appear in the low-density mononuclear cell fraction. However, Florcken et al. reported that there was no quantitative variation in MDSC levels between fresh unfractionated whole blood and PBMCs in patients with metastatic renal carcinoma [27]. This variation could be due to different factors. G-MDSCs were identified by their expression of vascular endothelial growth factor (VEGRF), while in our study, the cells were defined by their expression of CD33^dim^ and CD15 [27].

We found high levels of GMCs in whole blood, followed by APCs and MMCs, while IMCs showed the lowest level among the different myeloid cell subsets. In contrast, MMCs and APCs were highest in PBMC, while GMC and IMC were at low levels. These data showed that, indeed, the GMCs detected in PBMC comprise primarily of PMN-MDSCs, while in blood comprised mainly of mature neutrophils. Moreover, the levels of HLA-DR^−^ MMCs were overshadowed by GMCs in whole blood, but their levels in PBMCs represented their factual levels. These results prompted us to compare the levels of different circulating myeloid cells in PBMC between CRC patients and HDs. We did not find any statistical differences between levels of GMCs, IMCs, and APCs between CRC patients and HDs, although there was a trend of reduction in levels of circulating GMCs in CRC patients. It is noteworthy that these results could be due to several uncontrollable artifacts of the density-gradient isolation process such as cryopreservation and thawing of PBMCs. It has been shown to alter the phenotypical and functional characteristics of cells in that density-gradient fraction, particularly on the viability and recovery of G-MDSCs [28]. We used buffy coats from donors and fresh whole blood from patients for our investigations but isolated PBMC from both patients and donors, to mitigate any effects on GMCs. However, the difference in the starting material between CRC patients and HDs could have led to the relatively higher levels of GMCs in HDs than CRC patients, although statistically insignificant. Importantly, we found a significant increase in the levels of circulating MMCs in CRC patients. These MMCs comprise of suppressive M-MDSCs, as monocytes and M-MDSCs can be separated on the basis of expression of HLA-DR [9].

Tumor cells and neighboring stromal cells in the TME release various tumor-derived soluble factors that can promote the development and expansion of MDSCs [29]. Differences between circulating and tumor-infiltrating MDSCs have been previously described [29, 30]. In the TME, MDSCs acquire different characteristics compared with peripheral blood. In the former, MDSCs exert more suppressive actions as the M-MDSC cell subset and the classical activated monocytes (M1) quickly transform to TAMs, while some G-MDSCs transform to the highly suppressive tumor-associated neutrophils (TANs) [31]. Our results showed that the absolute count of GMCs was elevated in TT, compared to NT. However, MMCs along with the other myeloid cell subsets did not show any significant differences between NT and TT. The elevation in GMCs is consistent with our previous studies. However, previously, IMCs also showed a significant increase in TT compared to NT of CRC patients [25, 32]. The variability in findings could be due to enzymatic disaggregation performed previously to create a single-cell suspension from tissues, which can alter the expression of cell surface proteins [33].

Importantly, we investigated the differences between levels of myeloid cells in CRC patients with their clinicopathologic data. Patients with advanced disease stages showed significantly higher levels of GMCs compared to early stages in whole blood, but their levels in PBMCs and tumor-infiltrating myeloid cells did not show any significant differences. Circulating GMCs include mature neutrophils, and therefore, our results could indicate an elevated neutrophil to lymphocyte ratio (NLR) in patients with an advanced disease stage. NLR has been indicated as a marker for immune responses and importantly has been shown as a prognostic indicator in CRC patients [34, 35]. In addition, Zhang et al. previously showed correlations between increased levels of circulating Lin^−^HLA-DR^−^CD11b^+^CD33^+^ MDSCs in blood of CRC patients with disease staging and tumor metastasis [24]. We have also previously showed that expansion of peripheral GMCs in whole blood correlated with advanced disease stages and histological grading in CRC patients [32]. However, our comparisons of levels of GMCs in whole blood versus PBMCs in the present study highlight the significance of the abundance of granulocytes in whole blood, unlike PBMCs. In addition, tumor-infiltrating myeloid cell subsets, including GMCs, did not show any significant differences between disease stages in agreement with previous studies.

Tumor budding, identified by the manifestation of a solitary tumor cell or small aggregates or clusters of up to five tumor cells in the tumor stroma, is identified as a prognostic factor for CRC patients [36]. Meta-analysis of more than 4500 CRC patients showed association between tumor budding and lymph node metastasis [37]. Therefore, tumor budding scores are commonly recorded for CRC patients, especially those with early stages. In addition, LVI refers to invasion of tumor cells to surrounding blood vessels and/or lymphatics, and is considered as an important prognostic factor for earlystage CRC, which helps to determine the course of therapy [38, 39]. Our data indicated a trend of reduction in levels of tumor-infiltrating GMCs in patients with higher tumor budding scores, while IMCs showed an opposing trend. In contrast, we did not find any significant differences in the levels of different tumor-infiltrating myeloid cells in patients with LVI. These data could indicate the potential roles of different myeloid cell subsets in metastatic invasion and suggest other possible factors related to their functional activity, which warrant further investigations.

Myeloid cells are known to promote tumor growth by favoring angiogenesis, suppressing antitumor immunity, and promoting metastasis [40]. For instance, Ibrahim et al. showed that MDSCs affect STAT3 activation, which leads to silencing of tumor suppressor IRF8 in colonic epithelial cells, to promote colitis-associated colon tumorigenesis [41], while Lin et al. showed that liver metastasis in CRC patients could be initiated by MDSCs [42]. Our results indicate that the different myeloid cell subsets contribute to tumor progression in unique pathways since not all potential MDSC subpopulations were elevated in circulation or TME or associated with disease staging, tumor budding, or LVI in CRC patients. This heterogeneity and plasticity of immunosuppressive myeloid cells are also evident in tumor-bearing mice and in clinical trials on various human malignancies, which have shown inconsistencies in responses to therapy between circulating and tumor-infiltrating MDSC and make it challenging to target them [23]. Current therapeutic interventions developed to target MDSCs primarily are aimed at eliminating their suppressive factors, block recruitment and/or induce their depletion in the TME, and promote MDSC differentiation into mature cells [23, 43]. However, improving their selective targeting requires further preclinical and clinical investigations.

## 5. Conclusion

Immunosuppressive myeloid cells assist tumor progression via immune evasion and act as potential contributors of resistance for effective anticancer therapies. We investigated different myeloid cell subsets in periphery and TME of CRC patients. Our results showed that the levels of circulating myeloid cells were different between whole blood and PBMCs. Moreover, we found that circulating MMCs and tumor-infiltrating GMCs were elevated in CRC patients. Importantly, patients with advanced disease stages showed increased levels of GMCs in whole blood, but their levels were reduced in patients with high tumor budding. In contrast, levels of tumor-infiltrating IMCs were increased in patients with high tumor budding, suggesting their potential roles in metastasis and invasion. However, these findings warrant functional studies to ascertain their roles in disease progression.

## Figures and Tables

**Table 1:** Characteristic features of study populations.

	CRC patients	HDs
Number	88 (55^∗^, 31^∗∗^)	25^∗^
Age (median)	59 (18-96)^†^	32 (19-51)^†^
Gender (male : female)	58 : 30	19 : 6
TNM stage		
I	9 (7^∗^, 4^∗∗^)	
II	31 (18^∗^, 10^∗∗^)	
III	35 (21^∗^, 14^∗∗^)	
IV	13 (9^∗^, 3^∗∗^)	
Histological grade		
G2 moderately differentiated	All samples	
Tumor budding		
Low	13^∗∗^	
Intermediate	8^∗∗^	
High	10^∗∗^	
Lymphovascular invasion	16^∗∗^	

CRC: colorectal cancer; HDs: healthy donors. ^†^Median age. ^∗^Samples used for analyzing myeloid cells in PBMCs. ^∗∗^Samples used for analyzing myeloid cells in tissues.

## Data Availability

The data used to support the findings of this study are available from the corresponding author upon request.
